# Management of a Patient With Polypharmacy Toxicity (Calcium Channel Blocker, Angiotensin Converting Enzyme Inhibitor and Tricyclic Antidepressant) in Intensive Care Settings: A Case Report

**DOI:** 10.1155/cria/8424003

**Published:** 2026-05-31

**Authors:** Sami Ur Rehman, Faiza Zahid, Shaheryar Nazim, Caitriona Cody, Teasy Sweeney

**Affiliations:** ^1^ Department of Anaesthesia, Connolly Hospital Blanchardstown, Dublin, Ireland, connollyhospital.ie

**Keywords:** angiotensin-converting enzyme inhibitor (ACEI) toxicity, calcium channel blocker (CCB) toxicity, high insulin euglycemic therapy (HIET), tricyclic antidepressant (TCA) toxicity

## Abstract

We are presenting the case of a 28‐year‐old female patient who presented with refractory shock secondary to overdose of tricyclic antidepressants, calcium channel blockers (CCBs) and angiotensin‐converting enzyme inhibitors. After initial resuscitation, bicarbonate infusion for TCA toxicity was used; and CCB toxicity was managed with high‐dose insulin euglycemia therapy (HIET) with simultaneous dextrose infusion and vasopressors in the intensive care unit.

## 1. Introduction

Drug overdose toxicity remains a critical global health concern. The 2022 report of the American Association of Poison Control Centres states that the cardiovascular medications accounted for around 119,000 (or 4.74%) of all toxicity cases reported [1]. According to the health research board data published in September 2024, 354 drug poisoning deaths were recorded in Ireland in the year 2021, which equates to seven deaths per 100,000 of the population in that year. Opioids (69%), benzodiazepines (53%) and antidepressants (35%) were the three most common drug groups implicated in poisoning deaths overall [2]. This issue is not limited to illicit drugs but prescription medications (e.g., antidepressants and cardiovascular medications) are frequently implicated. Polypharmacy is widespread, increasing complexity in both prevention and treatment.

Tricyclic antidepressants (TCAs), although substituted by safer medications such as selective serotonin reuptake inhibitors (SSRIs), continue to be frequently prescribed for chronic pain. Sign and symptoms of TCA overdose are cardiac arrhythmias, central nervous system (CNS) depression, seizure activity and even comatose state, with significant mortality rates of 70%, if not managed actively [3]. Pathophysiology of TCA overdose includes inhibition of noradrenaline reuptake, serotonin reuptake and GABA receptors, leading to metabolic acidosis and mitochondrial dysfunction [3]. The therapeutic window of TCAs is narrow, so ingesting small doses like 10–20 mg/kg is life‐threatening. Symptoms of overdose usually starts in 30–40 min, and signs of toxicity are usually clinically revealed within 2 h, but delayed toxicity may occur.

Overdose of angiotensin‐converting enzyme inhibitors (ACEIs) usually does not cause haemodynamic instability; however, coingestion with other cardiovascular drugs can be linked with refractory shock and major metabolic disturbances [4].

Adverse effects of calcium channel blockers (CCBs) include vasodilatory effects such as peripheral oedema, dizziness, palpitation, flushing, decreased cardiac muscle contractility with reduced sinus pacemaker velocity and atrioventricular conduction [5].

We are presenting the case of a 29‐year‐old female with polypharmacy abuse of TCA, ACEI and CCB that is successfully managed in intensive care settings. CARE checklist is followed in writing down this case report.

## 2. Case Presentation

A 28‐year‐old female (weighing 75 kg with a height of 5 feet) presented to the Emergency Department following an intentional overdose of 24 tablets of amitriptyline (600 mg total) and 27 tablets of a combination of perindopril (135 mg total)/amlodipine (270 mg total). Her past medical history included autism spectrum disorder (ASD) and a documented history with psychiatric services for recurrent suicidal ideation and previous attempts.

On arrival, the patient was hypotensive with a mean arterial pressure (MAP) of 50 mmHg and heart rate (HR) of 110 beats per minute. Glasgow Coma Scale (GCS) score was 15, and oxygen saturation (spo2) was 95% on 3 L of supplemental oxygen. Initial arterial blood gas (ABG) analysis revealed severe metabolic acidosis with a pH of 7.27 and a markedly elevated lactate level of 9.6 mmol/L. All routine bloods (including FBC, Na, K, Ca, Mg, renal functions, liver functions, cardiac markers and PT/APTT/INR) were sent immediately and repeated every 6 hourly with serial 12‐lead ECG monitoring. Cardiology and internal medicine team were also kept in‐loop during all the management period. Immediate management involved securing central access, placement of an arterial line, goal‐directed fluid therapy, vasopressors/inotropes and urinary catheterization with target urine output more than 0.5 mL/kg/hour.

During this time, she was fully oriented and maintaining her airway with Spo2 > 94% at 5 L of oxygen with face mask. But given the severity of hypotension and early signs of shock, the patient required high vasopressor support, receiving high‐dose Noradrenaline (1 μg/kg/minute) alongside Vasopressin (0.04 units per minute) and dobutamine (10 μg/kg/minute). She also required adrenaline 10–20 μg IV boluses to maintain the target MAP of 65 mmHg.

Initially sodium bicarbonate was commenced to counteract the metabolic acidosis and potential TCA‐induced cardiotoxicity. After multidisciplinary discussion, TOXBASE was contacted for guidance on management and antidote recommendations. Following consultation with the TOXBASE U.K., the patient was started on the protocol for high‐dose insulin euglycemic therapy (HIET), a treatment primarily targeting CCB and beta‐blocker toxicity. The protocol commenced with a 50‐mL bolus of 50% glucose with a stat bolus of short‐acting insulin at 1 IU/kg, followed by short‐acting insulin infusion at 0.5 IU/kg/min, titrated every 30 min to a maximum of 10 IU/kg/hour based on haemodynamic response. Glucose was measured every 20 min for the first hour and hourly thereafter. Potassium was measured 1 hourly with the threshold of potassium replacement set at 2.5 mmol/L.

To prevent hypoglycaemia from the HIET, a dextrose 10% infusion was administered with blood glucose targets between 5.5 and 14 mmol/L. The patient also experienced an episode of coffee ground emesis, indicative of an upper gastrointestinal bleed. Despite the aggressive regimen, hypotension persisted overnight with increasing vasopressor requirements.

The vasopressors and inotrope requirement started decreasing second hour into the HIET and bicarbonate therapy. We started achieving therapeutic endpoints as per HIET after approximately 12 h of starting maximal inotropic and vasopressor support. Electrolyte and ABG monitoring were repeated hourly on the first day and then at 4‐h intervals throughout her ICU stay. Although there was an episode of tachycardia of up to 137/min with sinus rhythm reported at the time of ICU admission, continuous ECG monitoring on the cardiac monitor showed no signs of any arrythmias, QRS changes, PR interval lengthening or any segment changes thereafter, during the ICU stay period. Serial 12‐lead ECG monitoring was repeated every 4 h. All vasopressors and inotropes were stopped on 2^nd^ day of her admission, and HIET was also weaned after the removal of vasopressors. The dextrose infusion had to be continued for the next 12 h as her blood glucose levels were on the lower side (5‐6 mmol/L, with lowest blood glucose 4.9 mmol/L), even after stopping the HIET. In our case, potassium levels were well maintained within the normal range (3.5–5.5 mmol/L) during ICU stay without any need of potassium replacement. There was a rise in cardiac markers like Trop‐I and NT‐pro‐BNP on 1^st^ day but after being serial monitored, echocardiography, serial bedside POCUS and reviewed by cardiologist, it was concluded that this was due to very high doses of vasopressors being used.

**FIGURE 1:**
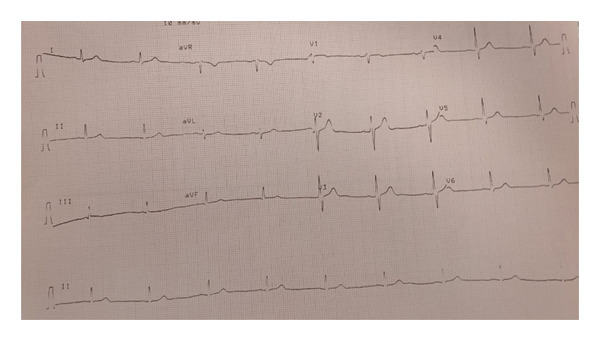
Electrocardiogram (ECG) of the current patient after hemodynamic disturbances got settled. This ECG was just before transfer from ICU to ward.

**FIGURE 2:**
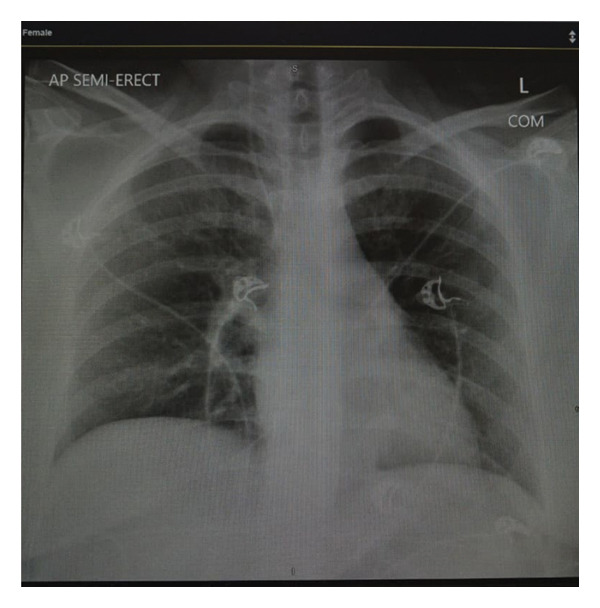
Chest X‐ray of the current patient with no significant finding.

**FIGURE 3:**
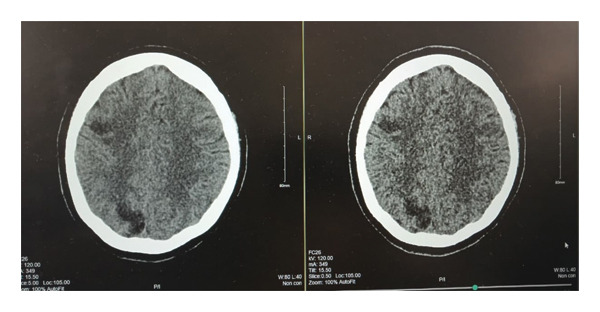
Computed tomography (CT) brain of the current patient showing a hypodense lesion approximately 2 ∗ 3 cm in right occipital lobe that is suspected suboccipital infarct. In right fronto‐occipital infarct, there is 2 ∗ 2 cm hypodense lesion suspecting an acute ischaemic infarct.

Several days into her recovery, she complained of new‐onset tingling and numbness on left thumb and lift upper lip. The patient had maintained a GCS of 15 throughout her ICU admission. Although she was on high vasopressor support, a total of 8 episodes of MAP < 65 mmHg occurred, each less than one minute in duration. A subsequent MRI of the brain revealed multiple ischaemic infarcts; it was done on Day 03 of ICU admission, i.e., on the next day of shock resolution. Figure 1 shows electrocardiogram at the time of discharge from hospital, Figure 2 shows Chest X‐ray at time of ICU admission and Figure 3 shows brain MRI findings. Evaluation was done to rule out embolic stroke of unknown origin including 24‐h Holter monitoring, transthoracic echocardiography and MRA of the brain. No significant finding in all the above tests was found that shows any embolic stroke. So, it was then concluded that it was either because of very high doses of catecholamines causing cerebral vasospasm or dips of MAP < 65 mmHg although those episodes never exceeded more than 1 min. Otherwise, she remained stable with no neurological deficits and improvement in tingling sensation after 3 days. She was already started on prophylactic subcutaneous enoxaparin 60 IU/day on 2^nd^ day of ICU as her PADUA prediction score for VTE is 4.

Psychiatry was engaged for assessment and protocol‐driven treatment of her underlying depression and suicidal ideation. Following a 3‐4‐day stay in the intensive care unit, the patient was discharged to the general medical ward for continued observation and psychiatric management. The patient has been deidentified for this publication (Table 1).

**TABLE 1:** LABS of the current patient from the time of admission in ICU to discharge from ICU.

LABS	On arrival in emergency department	ICU: 1^st^ bloods (Day 1) (9:45)	ICU: 2^nd^ bloods (Day 1) (16:00)	ICU: 3^rd^ bloods (Day 1) (22:30)	ICU: Day 02	ICU: Day 03	Day of discharge from ICU (Day 4)
Haemoglobin (g/dL)	13.6	12.1	10.8	11.1	10.7	9.7	9.6
Total leucocyte count (cells per mL)	25,000	26,800	20,400	26,200	26,500	16,300	9400
pH	7.37	7.34	7.38	7.36	7.42	7.44	7.41
Lactate (mmol/L)	9.6	10.44	3.62	2.38	1.85	1.39	0.96
C‐reactive protein (mg/L)	7.40	10.6	50.30	68.50	68.20	41.40	41
Creatinine (micromoles/L)	125	156	103	98	91	62	64
Sodium (mmol/L)	140	140	145	144	142	141	144
Potassium (mmol/L)	3.9	4.0	4.1	4.0	4.1	4.7	3.7
Calcium (mmol/L)	2.33	2.10	2.09	2.10	2.12	2.11	2.08
CPK (units/L)	—	193	504	—	321	117	—
Blood glucose (mmol/L)	10.3	12.2	7.7	5.9	6.2	5.8	5.6
Magnesium (mmol/L)	1.10	1.02	0.76	1.39	1.06	0.85	0.87
Troponin‐T (ng/mL)	< 45.000	< 45.000	479.140	738.240	512.32	242.56	< 45.00
NT‐Pro‐BNP (pg./mL)	—	100	—	5113	4892	1286	321

## 3. Discussion

While managing polypharmacy overdose who rapidly developed severe refractory shock, multimodality support is always required. Deciding what agent is the culprit for cardiovascular instability in such cases is very important in planning management strategy. In our case, patient had overdosed three drugs with known cardiac side effects: amlodipine, amitriptyline and perindopril. So, this case is unique because there is no such literature of these groups of drugs all together and our patient recovered quite early with normal mentation (GCS 15/15) throughout and no respiratory abnormality.

Half‐life of amlodipine is around 30–58 h [6]. Being a dihydropydine CCB, it has more affinity for vascular smooth muscles rather than myocardium. In literature, cases have been described with anoxic brain injury with doses of up to 80 mg, and a favourable outcome even with high dose of about 350 mg [7].

The important decision‐making challenge was to decide whether to start HIET early or wait for the response to vasopressors and fluid therapy. But after the multidisciplinary communication between intensivist, emergency medicine consultant and internal medicine, it was decided to contact TOXBASE to start the early HIET because apart from experience, there are multiple case reports available with better outcome in early HIET vs. late HIET after all other therapies fail [8, 9].

Perindopril is a prodrug with a half‐live of 17 h, which influences blood pressure 2–5 h after ingestion. This occurs because of the inhibition of normal homeostatic mechanism of RAAS activation in response to shock. Other than above causes, ACEIs result in bradykinin accumulation, which then causes vasodilation and resultant hypotension. Although we didn’t opt for any specific antidote for ACEI but in the literature, naloxone IV (as bolus up to 2 mg and then as infusion) is practiced in case of severe bradycardia secondary to ACEI overdose [10].

The adverse effects of TCA include risk of seizures, metabolic acidosis, coma and the characteristic QRS prolongation seen in TCA overdose, which can lead to heart block and bradycardia, and in some cases, may cause torsade’s de pointes. TCA has a known quinidine‐like effect on myocardium causing decreased cardiac contractility and resultant hypotension [2, 3].

Initial management of such polypharmacy overdose is to ensure clean airway, securing airway if indicated and supportive measures to maintain haemodynamic with intravenous fluids, vasopressors like noradrenaline and vasopressin because CCB and ACEI toxicity cause vasoplegia and in case of suspected myocardial dysfunction, dobutamine can be used. Then comes the specific management, which is explained for each agent in the following. Also, sending serial electrolytes, arterial blood gas analysis and continuous monitoring of vital signs and cardiac rhythms is crucial. Getting a transthoracic echocardiography and chest X‐ray and serial 12‐lead ECG is also an integral part of this management. CT‐brain or MRI‐brain only of indicated as in our case after the haemodynamic are stable. Drug specific antidote management is given in the following in each paragraph.

As per current TOXBASE guidelines, the first approach for TCA overdose includes sodium bicarbonate (NaHCO3) infusions along with supportive measures used to reduce the toxicokinetic effects by keeping the pH towards the alkalotic side (7.45–7.55). NaHCO3 competitively inhibits the binding of TCAs to cardiac sodium channels and ultimately helps restoring normal electrical conduction in the heart [11].

As per TOXBASE, high‐dose insulin‐euglycemic treatment regimen (HIET) has been used successfully to improve circulatory stability in patients with CCB overdose. As per this protocol, start with 50% of 50 mL glucose (25 grams glucose) unless blood glucose more than 22 mmol/L and then bolus of short‐acting insulin bolus at 1 IU/kg, followed by short‐acting insulin infusion at 0.5 IU/kg/hour with 30 min assessment, and this can be titrated up to 5 IU/kg/hour, along with dextrose infusion at 25 g/hour [5, 12]. Blood glucose monitoring is recommended every 20 min for first hour and then 1 hourly with a target of 5.5–14 mmol/L and same for potassium monitoring at 1 hourly rate via arterial blood gas analysis, with the aim of not correcting hypokalaemia until and unless it is less than 2.5 mmol/L, as this drop in potassium is the direct result of insulin effect on intracellular displacement of potassium from the extracellular space [12]. This increased intracellular potassium causes increased calcium inflow into the cells which helps with myocardial contractility and helps stabilize the cardiac muscle cell membranes [13]. The most common adverse effects of HIET are hypoglycaemia, hypokalaemia, hypomagnesemia and hypophosphatemia. The therapeutic endpoint is the same as already explained in case presentation part with EF > 50%, SBP > 90 mmHg, improved mentation, improved acidaemia and removal of cardiac conduction abnormalities, and HIET is weaned after the withdrawal of vasopressors.

Our patient responded with HIET but as per literature, in case of refractory hypotension and periarrest situations, second‐line therapy for CCB toxicity can be started that is intralipid infusion with the recommended dose of 1–1.5 mL/kg as a bolus and then 0.25 mL/kg/minute for 60 min [14]. In literature, there was also use of calcium infusions as 10–20 mL (1‐2 g) every 10–20 min or an infusion rate of 0.2–0.4 mL/kg/h in the form of 10% calcium chloride. If 10% calcium gluconate is used instead, the dose would be 30–60 mL (3–6 g) every 10–20 min or an infusion rate of 0.6–1.2 mL/kg/h [15]. If all of the above strategies are ineffective, extracorporeal life support can be employed to maintain organ perfusion. ECMO has been successfully used to support patients with CCBs toxicity [16].

As our patient had started responding to vasopressors and goal‐directed fluid therapy after HIET and bicarbonate infusion, we did not opt for venoarterial extracorporeal membrane oxygenation. But in the case of refractory shock, we would recommend early initiation of ECMO [6]. The suggested criteria for ECMO initiation in CCB‐related shock include persistent cardiac dysfunction despite catecholamine therapy (LVEF < 40–50%), high‐dose vasopressor requirements (epinephrine + norepinephrine > 3 mcg/kg/min) and elevated blood lactate concentration (> 8 mmol/L) [17].

While haemodynamic stability was achieved, the delayed neurological sequelae serve as a stark reminder of the devastating consequences of prolonged refractory shock. Ongoing psychiatric care remains paramount for addressing the underlying causes of the overdose. The success of such polypharmacy toxicity management cases is early initiation of aggressive, multimodal critical care and specialized toxicology protocols, and in cases of refractory shock, the timely initiation of ECMO.

## 4. Conclusion

Managing a multidrug toxicity of CCB, ACEI and TCA consist of airway management, supportive measures to maintain haemodynamic (fluids, vasopressors and inotropes), monitoring of vital signs, cardiac rhythms and specific management of drug toxicity like bicarbonate infusion for TCA, HIET for CCB and Angiotensin II or naloxone for ACEI only in cases with refractory shock. ECMO can be utilized in patients not responding to pharmacological treatment.

### 4.1. Learning Points for Anaesthesiologists

1. Initial management of polypharmacy overdoses is to ensure clean airway, securing airway if indicated and supportive measures to maintain haemodynamic with intravenous fluids and vasopressors.
2. Starting HIET earlier for CCB toxicity has better outcomes. As per this protocol, start with 50% of 50 mL glucose (25 grams glucose) unless blood glucose more than 22 mmol/L and then bolus of short acting insulin bolus at 1 IU/kg, followed by short‐acting insulin infusion at 0.5 IU/kg/hour with 30 min assessment, and this can be titrated up to 5 IU/kg/hour, along with dextrose infusion at 25 g/hour.
3. For TCA overdose includes sodium bicarbonate (NaHCO3) infusions along with supportive measures used to reduce the toxicokinetic effects by keeping the pH towards the alkalotic side (7.45–7.55).
4. Serial electrolytes, arterial blood gas analysis and continuous monitoring of vital signs and cardiac rhythms are crucial. Getting a transthoracic echocardiography and chest X‐ray and serial 12‐lead ECG is also an integral part of this management.
5. In case of refractory shock, we would recommend the early initiation of ECMO. The suggested criteria for ECMO initiation in CCB‐related shock include persistent cardiac dysfunction despite catecholamine therapy (LVEF < 40–50%), high‐dose vasopressor requirements (epinephrine + norepinephrine > 3 mcg/kg/min) and elevated blood lactate concentration (> 8 mmol/L).

## Author Contributions

Sami Ur Rehman: conception of study design and original draft writing.

Faiza Zahid: literature review.

Shaheryar Nazim: writing the case presentation part.

Caitriona Cody: proofread.

Teasy Sweeney: proofread.

## Funding

No funding was sorted for the publication of this case report.

## Consent

Informed consent was obtained from the patient for publication of this case report. There is no need of ethical approval letter from institution as per health services executive research committee guidelines as this is includes only one case.

## Conflicts of Interest

The authors declare no conflicts of interest.

## Data Availability

Research data are not shared.
